# A new test for evaluation of marginal cognitive function deficits in idiopathic normal pressure hydrocephalus through expressing texture recognition by sound symbolic words

**DOI:** 10.3389/fnagi.2024.1456242

**Published:** 2024-09-17

**Authors:** Chihiro Kamohara, Madoka Nakajima, Yuji Nozaki, Taiki Ieda, Kaito Kawamura, Kou Horikoshi, Ryo Miyahara, Chihiro Akiba, Ikuko Ogino, Kostadin L. Karagiozov, Masakazu Miyajima, Akihide Kondo, Maki Sakamoto

**Affiliations:** ^1^Research Institute for Diseases of Old Age, Juntendo University School of Medicine, Tokyo, Japan; ^2^Department of Neurosurgery, Juntendo University School of Medicine, Tokyo, Japan; ^3^Department of Informatics, Graduate School of Informatics and Engineering, The University of Electro-Communications, Tokyo, Japan; ^4^Department of Neurosurgery, Saiseikai Kawaguchi General Hospital, Saitama, Japan; ^5^Department of Neurosurgery, Juntendo Koto Geriatric Medical Center, Tokyo, Japan

**Keywords:** sound symbolic words, texture recognition, idiopathic normal pressure hydrocephalus, dementia, neuropsychological test

## Abstract

**Introduction:**

The number of dementia patients is increasing with population aging. Preclinical detection of dementia in patients is essential for access to adequate treatment. In previous studies, dementia patients showed texture recognition difficulties. Onomatopoeia or sound symbolic words (SSW) are intuitively associated with texture impressions and are less likely to be affected by aphasia and description of material perception can be easily obtained. In this study, we aimed to create a test of texture recognition ability expressed by SSW to detect the presence of mild cognitive disorders.

**Methods:**

The sound symbolic words texture recognition test (SSWTRT) is constructed from 12 close-up photos of various materials and participants were to choose the best SSW out of 8 choices to describe surface texture in the images in Japanese. All 102 participants seen in Juntendo University Hospital from January to August 2023 had a diagnosis of possible iNPH (age mean 77.9, SD 6.7). The answers were scored on a comprehensive scale of 0 to 1. Neuropsychological assessments included MMSE, FAB, and the Rey Auditory Verbal Learning Test (RAVLT), Pegboard Test, and Stroop Test from the EU-iNPH Grading Scale (GS). In study 1 the correlation between SSWTRT and the neuropsychological tests were analyzed. In study 2, participants were divided into two groups: the Normal Cognition group (Group A, *n* = 37) with MMSE scores of 28 points or above, and the Mild Cognitive Impairment group (Group B, *n* = 50) with scores ranging from 22 to 27 points, and its predictability were analyzed.

**Results:**

In study 1, the total SSWTRT score had a moderate correlation with the neuropsychological test results. In study 2, there were significant differences in the SSWTRT scores between groups A and B. ROC analysis results showed that the SSWTR test was able to predict the difference between the normal and mildly impaired cognition groups.

**Conclusion:**

The developed SSWTRT reflects the assessment results of neuropsychological tests in cognitive deterioration and was able to detect early cognitive deficits. This test not only relates to visual perception but is likely to have an association with verbal fluency and memory ability, which are frontal lobe functions.

## 1 Introduction

As the population of elderly individuals continues to grow, so does the number of individuals diagnosed with dementia. Therefore, it is essential to identify the early signs of dementia in order to ensure that patients receive the appropriate treatment such as preventive training or medication. Although the mini-mental state examination (MMSE) (Folstein et al., 1975) is the most commonly used cognitive screening test, it is important to note that such tests must be administered by trained specialists, primarily neuropsychologists. However, in Japan, there are relatively few of them, and they are often employed in a part-time job basis, which results in a shortage of patients’ needs for professional assessment (Sakamoto, 2016). Additionally, more detailed neuropsychological tests can be a burden for some patients since they are often lengthy and require patients’ active participation. Some patients decline to take the cognitive tests due to embarrassment at making mistakes and confronting their declined cognitive abilities. Consequently, it is imperative that to develop a cognitive screening test that is simple and easy to administer, the patients feel comfortable to take it and it is sensitive enough to detect the early stages of dementia.

Many tests have been developed to detect the cognitive decline of people with dementia. However, only a few focused on the ability of texture recognition where it would be essential for daily living. In our living, it is necessary to estimate the state of the object from just looking at it. When you see a floor wet with water and seems slippery, then you would walk carefully not to slip. But when you fail to detect such slipperiness from a lack of texture recognition ability, then you would fail to take careful steps. Oishi et al. (2020), for example, focused on the ability to detect vegetable freshness through texture recognition and those with Lewy bodies had significantly impaired vegetable freshness perception (Oishi et al., 2020). In other previous research, disparities in cognitive ability related to the recognition of texture in presented images have been documented between individuals with Lewy bodies and Alzheimer’s diseases as well, compared to those without them (Oishi et al., 2020; Oishi et al., 2018; Battelli et al., 1997; Cavina-Pratesi et al., 2010; Bassi et al., 1993). Individuals with these diseases exhibited difficulty differentiating wet and shiny objects when shown in a picture.

Although the relationship between the sound of a word and its meaning has long been considered to be arbitrary (e.g., Saussure, 1916), the existence of synesthetic associations between sounds and sensory experiences (sound symbolism) has been demonstrated over the decades (Jespersen, 1922; Köhler, 1929; Newman, 1933; Ramachandran and Hubbard, 2001; Sapir, 1929; Taylor, 1963; Werner and Wapner, 1952; Wertheimer, 1958) and to a varying extent in a wide variety of languages (Brown et al., 1955; Emeneau, 1969; Hinton et al., 1994; Klank et al., 1971; Nuckolls, 1999; Voeltz and Kilian-Hatz, 2001). The majority of research on sound symbolism has tended to focus on cross-modal correspondences between speech sounds and visual shapes as demonstrated by the landmark studies of Köhler (1929) and Ramachandran and Hubbard (2001), which showed that nonsense words such as “malma” and “bouba” tend to be associated with rounded shapes, while nonsense words such as “takete” and “kiki” tend to be associated with angular shapes. It has been claimed that this systematic association between shapes and words is observed across diverse linguistic and cultural backgrounds (Wong et al., 2022). Also, previous research indicates that the age differences have no impact on the use and selection of SSW, particularly among individuals above the age of 11 (Sapir, 1929; Stanley et al., 2019; Maurer et al., 2006).

Recently, several studies have shown the association between sound symbolic sounds and tactile texture (Dingemanse, 2012; Kagitani et al., 2014; Sakamoto and Watanabe, 2018; Dingemanse and Majid, 2012). Sound symbolic words (SSW) or commonly called as onomatopoeia, are those in which the auditory information from the environment is verbalized. There are a larger number of SSW in Japanese compared to other languages (Sakamoto and Watanabe, 2018), and these Japanese SSW are commonly used from the early stages of language acquisition (Fernald and Morikawa, 1993). The previous study by Hashimoto (2014) indicated that SSW were used more often by patients with aphasia than by healthy individuals, and apparently they were less likely to be affected by symptoms of aphasia. Also, Oishi (2024) mentions how onomatopoeia or SSW would help identify and characterize the properties of the indicated materials (Oishi, 2024). Therefore, using the SSW to describe the texture shown in the photos would be suitable.

Among the various types of dementia, idiopathic normal pressure hydrocephalus (iNPH) is characterized by a decline in cognitive function that can be treated through Cerebrospinal Fluid (CSF) shunt surgery. Individuals with iNPH typically present with symptoms beginning at 60 years of age or older and have three main symptoms: gait and balance disturbance, urinary incontinence, and cognitive deterioration. The diagnosis of iNPH is made according to the flowchart in the Japanese iNPH Guidelines (Nakajima et al., 2021). At each step, the diagnosis is labeled as either possible, probable, or definite iNPH. For the cognitive impairment, it is often reported that there are dysfunctions in psychomotor speed, attention, working memory, and verbal fluency, as well as executive functions which are mostly frontal-lobe-related functions (Nakajima et al., 2021). Hellstrom et al. (2007) have indicated that compared to healthy individuals, iNPH patients perform worse in tests of learning and memory, manual dexterity, psychomotor speed, and executive function. Considering the results, Hellström et al. (2012) created an EU-iNPH grading scale to assess the efficacy of treatment interventions, the CSF shunt surgery, and the neuropsychological tests were successful in evaluating the changes after the treatment (Hellström et al., 2012). The neuropsychological tests include the Grooved Pegboard test, the Rey Auditory Verbal Learning Test (RAVLT), two tasks of the Stroop tests, the color naming, and the interference tasks (Hellström et al., 2012).

In this study, we aimed to create a test to detect the cognitive decline in patients with iNPH by analyzing the texture recognition of an image using SSW. We expected the test would be capable of detecting less pronounced cognitive decline with a shorter testing time, simple instructions, and less patient load than existing neuropsychological tests such as the MMSE. We have hypothesized that those with mild cognitive deterioration would have difficulty in selecting SSW to describe texture in an image, whereas those who maintain their cognitive ability at nearly healthy levels would not have such difficulty. For the need of the comparison of cognitive ability, individuals were evaluated using the EU-iNPH grading scale for iNPH patients as it has unified methodology and adequate sensitivity in diagnosing mild cognitive deficits.

## 2 Methods

### 2.1 Participants

We recruited 102 consecutive patients referred from all around Japan who visited Juntendo University Hospital and Juntendo Tokyo Koto Geriatric Medical Center, from January to August 2023 and were diagnosed as possible iNPH by either neurosurgeons or neurologists according to the Japanese iNPH Guidelines (Nakajima et al., 2021). This research consists of two separate studies in which in study 1 all patients were evaluated by the neuropsychological tests of the EU-iNPH grading scale as an established “golden” standard and compared to the newly created texture recognition test. Next in study 2, 87 of the same patients were diagnosed as probable or definite iNPH and additionally analyzed.

### 2.2 Sound symbolic words texture recognition test

The SSWTRT was developed using a distinctive data set comprising 1,000 original photographs which were initially collected for this study. All test images were generated by capturing close-up images of various object surfaces. The rationale behind opting for close-up images lies in the primary objective of evaluating participants’ ability to recognize and express what would be the object surface texture. Conversely, more distant images of whole objects may prompt participants to infer textures based on past experiences associated with the recognized object type rather than discerning the presented object’s surface texture. For instance, if a participant identifies a blanket, their inference of texture may rely more on past experiences with blankets than on the presented blanket’s surface.

The images were resized to 150 × 150 pixels by cropping the focused parts of the object. The dataset comprises materials from 10 categories (fabric, foliage, glass, leather, metal, paper, plastic, stone, water, wood), with 100 images prepared for each category. These categories were chosen in alignment with the “Flickr Material Database” (Sharan et al., 2014) a repository of material images featuring common textures.

**Figure 1:**
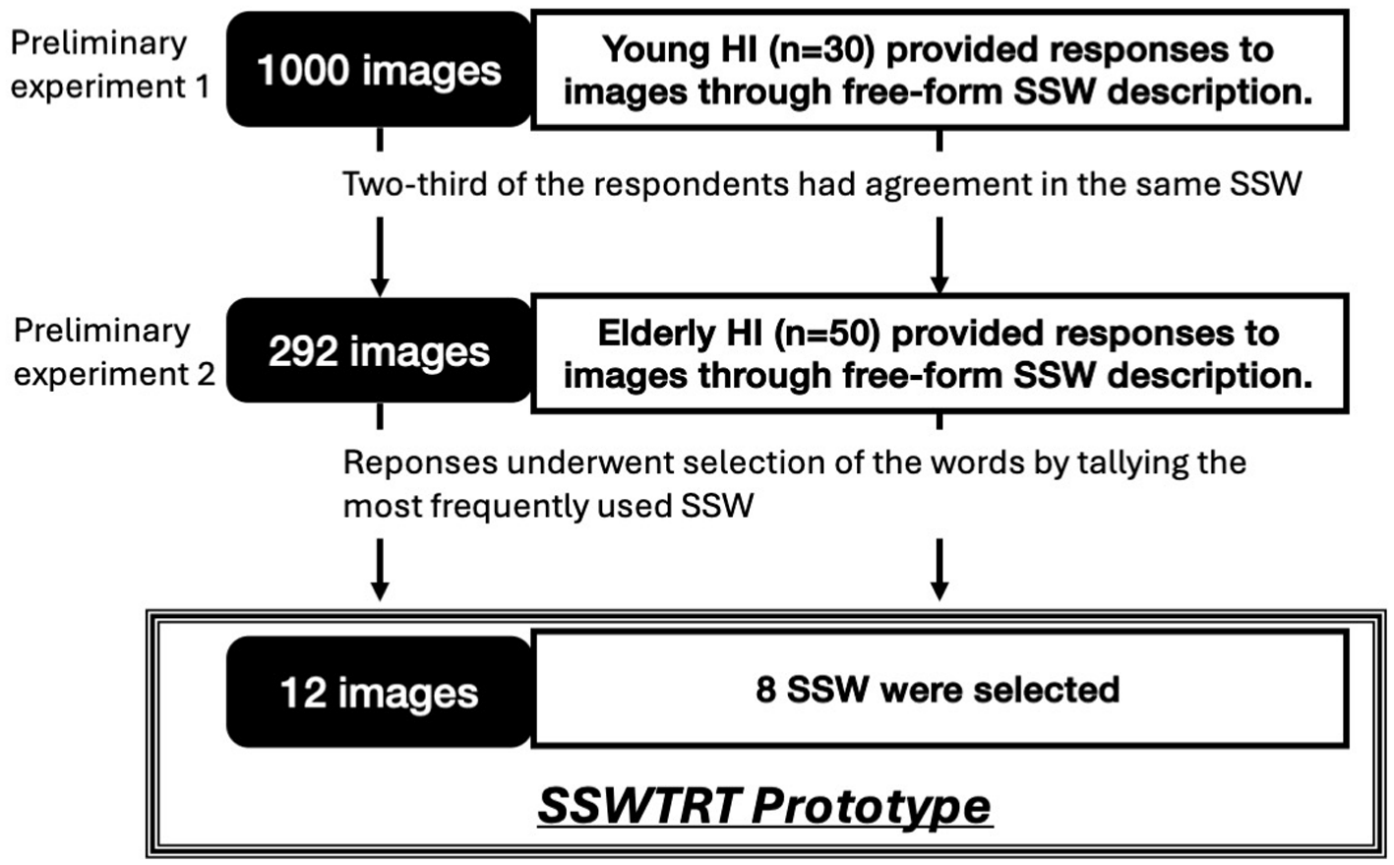
Flow chart of preliminary experiments and studies 1 and 2. HI, Healthy individuals; SSW, Sound symbolic words; SSWTRT, Sound symbolic words texture recognition test. This flow chart indicates the process of two preliminary experiments done to create the SSWTRT. This flow chart indicates the process of two preliminary experiments done to create the SSWTRT prototype and the two studies to comparatively evaluate the SSWTRT with the neuropsychological tests. In the first preliminary test, 1,000 images that we had originally collected were divided to a subset of 5, 200 images included in each subsets, and were shown to young healthy individuals. The participants were told to answer the texture perception through free-form SSW description. From their answers, 292 images in which 2/3 of the participants agreed on using the SSW were extracted. In the second preliminary experiment, those 292 images were shown to elderly healthy individuals and they were asked to do the same as the young individuals. According to the data obtained from the elderly healthy individuals, eight onomatopoeia or SSW were extracted and 12 images were chosen as the items included in the SSWTRT prototype.

**Figure 2:**
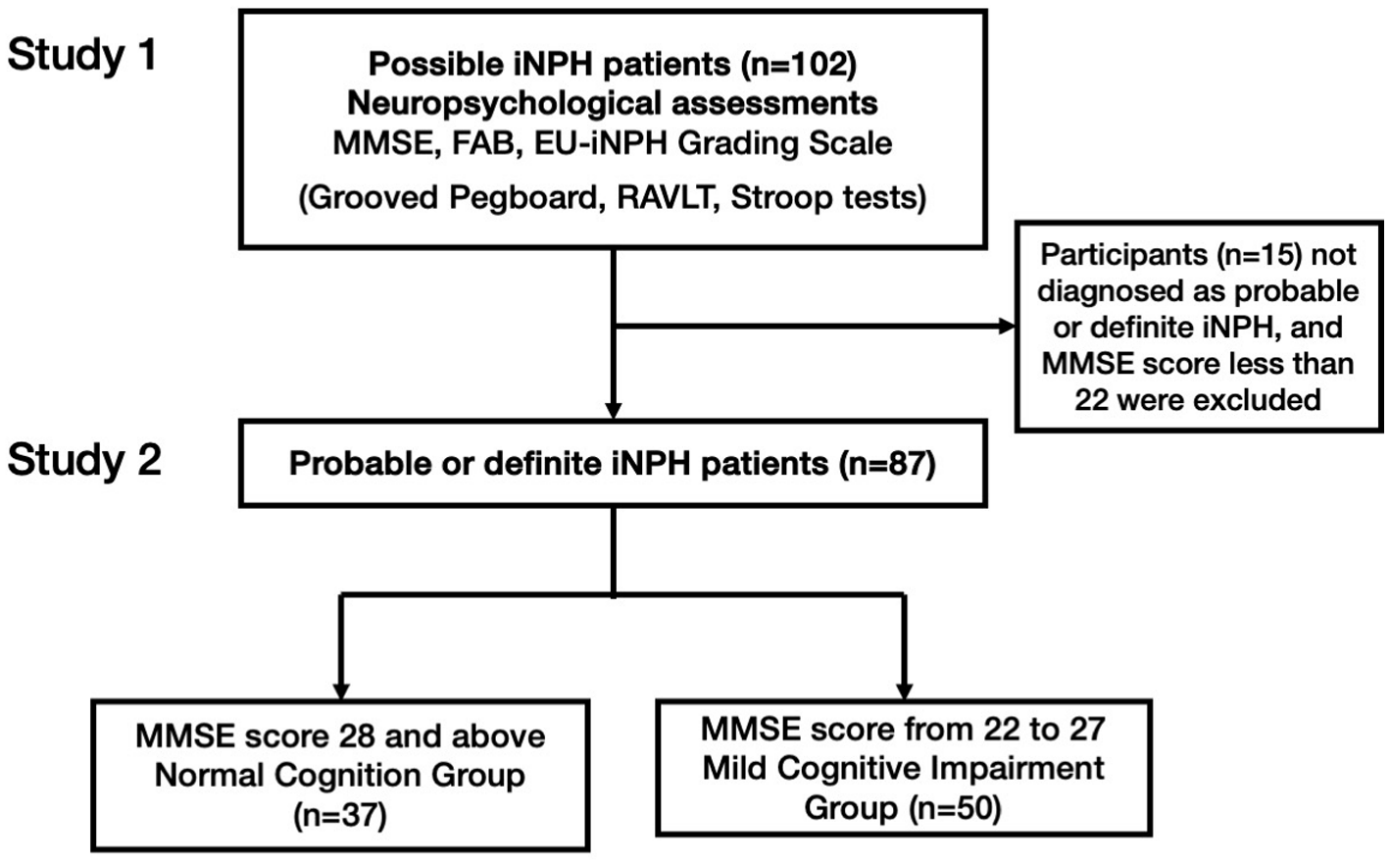
The flow chart of the study method. RAVLT: the Rey auditory verbal learning test; iNPH, idiopathic normal pressure hydrocephalus; MMSE: Mini-mental state examination; FAB: Frontal lobe assessment battery. In study 1, participants diagnosed as possible iNPH underwent SSWTRT and MMSE, FAB, and four tests from EU-iNPH Grading Scale (Grooved Pegboard, RAVLT, Stroop tests). Then in study 2, those who were diagnosed as probable or definite iNPH were separated according to MMSE test scores and its predictability was analyzed.

To identify images suitable for the texture recognition test and explore an appropriate set of SSW choices, two preliminary experiments were conducted which is shown in Figure 1. The initial preliminary experiment aimed to identify images suitable for the cognitive test from a pool of 1,000 images and was conducted on young, healthy individuals. It is essential for images used in cognitive assessments, before applied to the elderly, to yielded consistent responses while applied to young, healthy individuals. Images eliciting divergent responses among healthy subjects, who are presumed to possess similar texture recognition abilities, are considered to be significantly influenced by cultural and experiential factors. Consequently, such images were deemed unsuitable for inclusion in the assessment test (Figure 2).

To conduct this preliminary experiment, 30 participants (27 males, three females, average age = 22.27 years, SD = 0.94), native Japanese speakers, were grouped into five panels for evaluation. A 1,000 images reflecting texture were randomly divided into five subsets, each containing 200 images. Each panel was assigned one image set for the evaluation test. Images sized at 5 cm by 5 cm were presented to participants on a tablet device’s liquid crystal display (LCD) screen. Participants provided responses to the texture of their assigned set of 200 images using free-form SSW descriptions.

These SSW responses were subsequently quantified into an adjective scale following the methodology of previous studies (Sakamoto, 2020). From the initial pool, we selected 292 images where 2/3 of the respondents in the group concurred on the impression value direction of the texture recognition (positive or negative) across six scales representing the basic perceptions characteristics of texture by touch, for example: “smooth-rough,” “bumpy-flat,” “warm-cold,” “wet-dry,” “slippery-sticky,” and “hard-soft” (Doizaki et al., 2017).

The second preliminary experiment aimed to further refine the image selection and explore suitable choices. It was important for responses to exhibit consistency among a group of healthy elderly individuals. Although this condition is expected to align with the criteria applied to young and healthy individuals, conducting an extensive image examination poses physical challenges for the elderly. Consequently, the limited number of images (292) preselected through the experiment on young individuals were assessed to determine if they met this criterion. Fifty healthy elderly participants (25 men, 25 women, average age 78.6 years, SD = 2.99), native Japanese speakers, aged between 75 and 90 and without visual impairments, took part in the experiment. Similar to the experiment involving young individuals, participants described the texture of presented images using free-text SSW on a tablet device.

**Figure 3:**
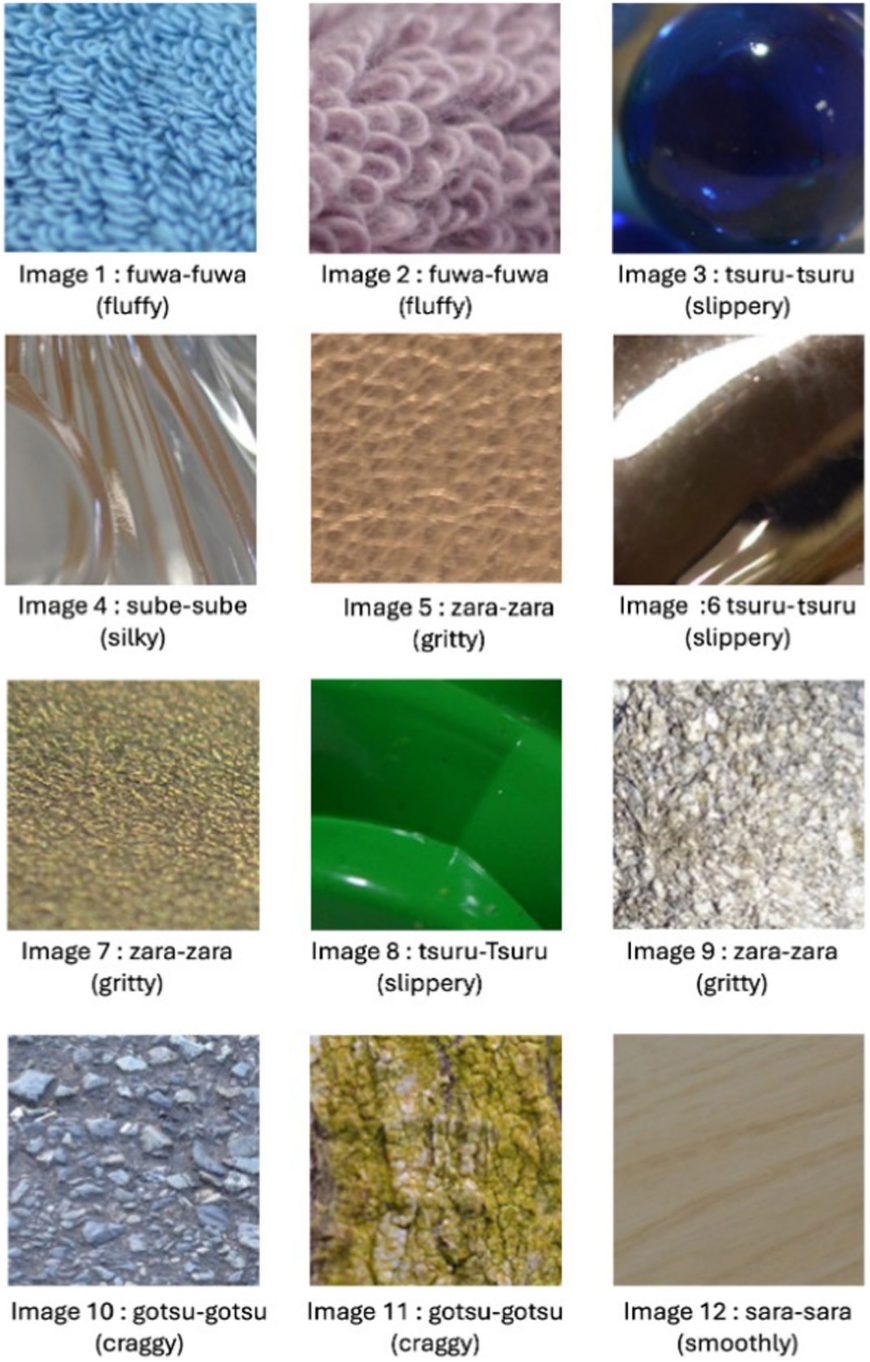
The images with matched sound symbolic words. This figure indicates the 12 images used in the sound symbolic word texture recognition test (SSWTRT). The sound symbolic word (SSW) shown below each image are SSW or onomatopoeic expressions that healthy elderly individuals would use according to the data we obtained in our preliminary research.

**Figure 4:**
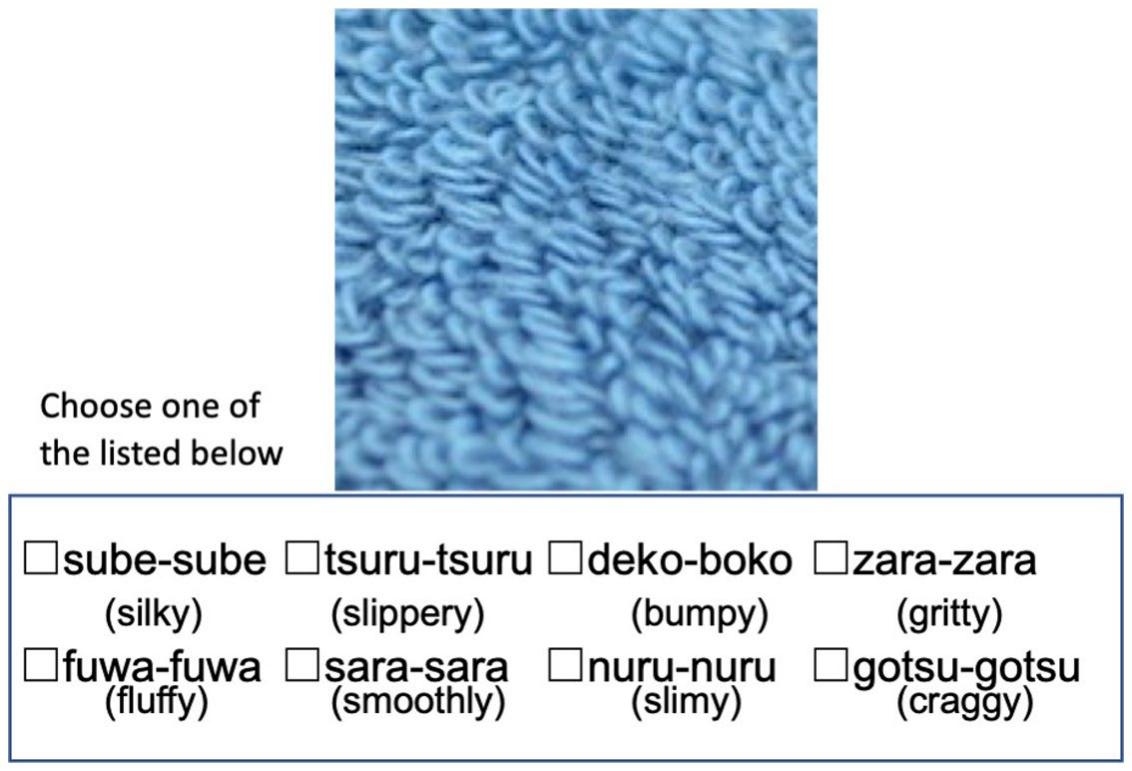
The sound symbolic words texture recognition test (SSWTRT). Each A4-sized sheet contains four images, each sized at 5 cm by 5 cm, with eight types of onomatopoeia or SSW, serving as answer options, listed beneath each image. The response sheets are grouped in sets of four, with the sequence of the 12 images and the order of onomatopoeic responses randomized within each set. The participants are asked to answer how the patients would describe the tactile recognition evoked by watching the image provided, choose the answer from eight choices of SSW and mark a check. The questions were provided after explaining what SSW meant and given an example question. The photos and onomatopoeia words are shown randomly, and the participants are free to start with any questions. The English-translated words are added just for this paper.

In the process of selecting SSW for the cognitive ability test, we identified 8 frequently used onomatopoeic expressions based on the responses obtained in this preliminary experiment with the elderly. This selection was made by tallying the occurrences of all onomatopoeic expressions used in the responses. The images where the eight most frequently used SSW were selected for the cognitive ability test. From them, a total of 12 images were ultimately chosen, which included fabric, glass, leather, metal, plastic, stone, and wood. Table 1 indicates the selected eight onomatopoeia words and their meanings. Figure 3 shows the 12 images along with the onomatopoeic expressions that were frequently used in response to each image. In the test sheet, each A4-sized sheet contains four images, each sized 5 cm by 5 cm, with eight types of SSW, serving as answer options, listed randomly beneath each image. The response sheets are grouped in sets of three, with the sequence of the 12 images and the order of onomatopoeic responses randomized within each set. The test created through these procedures is named the Sound Symbolic Words Texture Recognition Test or SSWTRT. Figure 4 illustrates an example of an answer sheet designed for the test. The participants were asked to evaluate “What texture would you expect to feel if you were able to touch the material shown in the photo” and choose the answer from eight choices of SSW. Prior to the administration the participants were provided with an explanation of the SSW by the examiner. In the description, the term “Onomatopoeia” is used to indicate SSW and examples questions are given to the participants to see how well they understand the SSW and the instruction of the test.

**Table 1:** Selected sound symbolic words and their meanings.

Sound symbolic words	Meaning
zara-zara	A texture and overall appearance that is coarse and has a strong roughness
tsuru-tsuru	A surface that is flat and glossy. A state of being smooth. This word is frequently used for hard materials like boards or metals.
fuwa-fuwa	Softly swollen or puffed up in appearance
sara-sara	Lacking moisture or stickiness
gotsu-gotsu	Angular and hard in appearance. Not flexible or supple.
sube-sube	A texture and appearance that is smooth and pleasing to the touch. The condition of skin or hair being smooth when touched.
nuru-nuru	A slimy and slippery state, causing discomfort, as if something mucous-like is clinging.
deko-boko	A surface that is not flat, having bumps and indentations.

### 2.3 Scoring

Scoring of the test was carried out by comparing participant’s responses to the distribution of the group of healthy participants. In other words, our scoring method gives a higher value when the chosen option is more frequently used by the healthy participant group.

**Figure 5:**
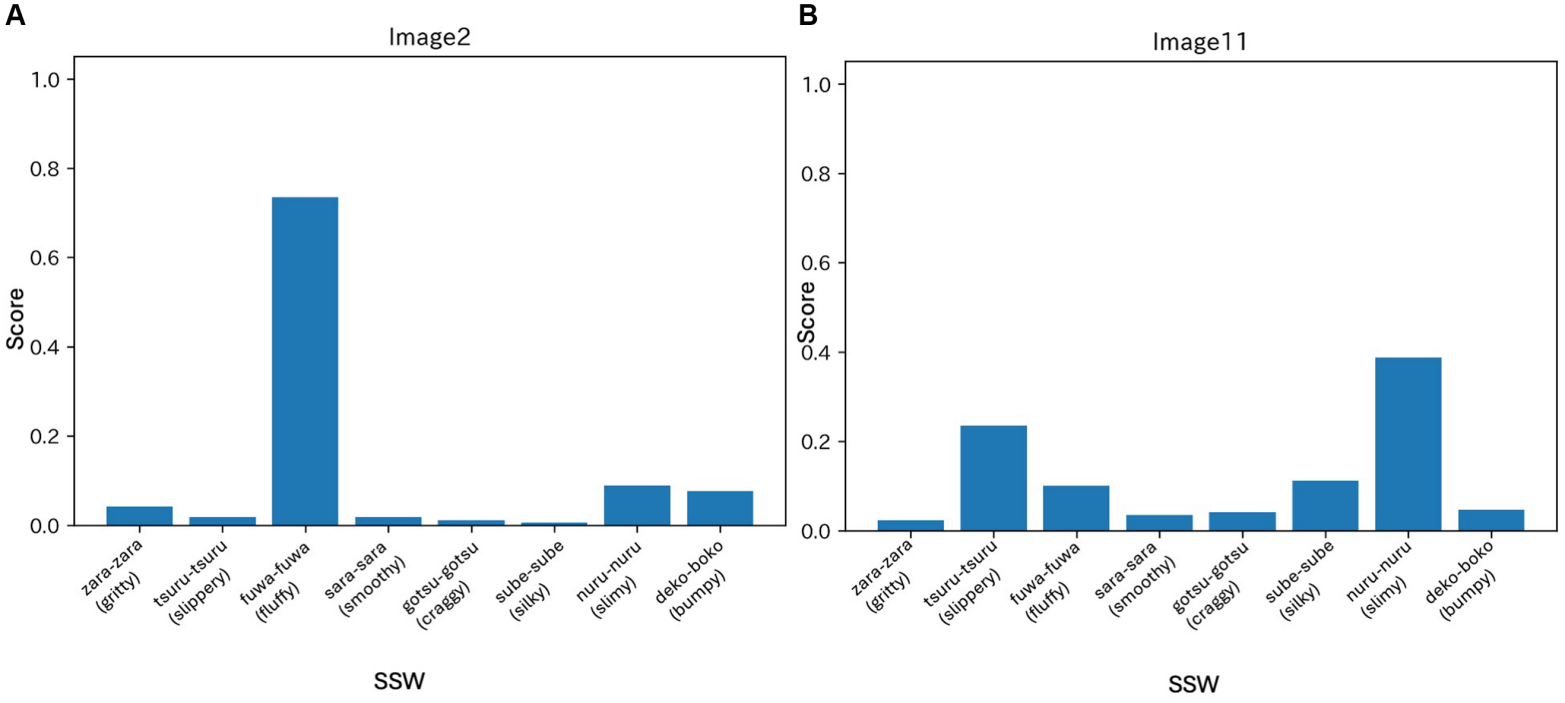
The choices of the most frequently used SSW to describe texture according to the preliminary experiments. **(A)** Indicates the result of the university students answering the SSW of image 2. It shows that 91.3% of participants chose the SSW “fuwa-fuwa” as the texture that they recognized in the image. This would lead to a high score in this study when the elderly participants chose their SSW to describe the texture of the image 2. **(B)** Indicates the result of young participants choosing the best SSW to describe the texture that they recognized for image 11. The SSW choices of “tsuru-tsuru” and “nuru-nuru” were selected in 29.0 and 46.4%, respectively. The SSW “tsuru-tsuru” indicates the smoothness of the surface, where “nuru-nuru” indicates the slippery texture.

To collect the response distribution of the healthy participant group, a questionnaire survey was conducted in June 2023 as part of a lecture with 69 students (57 men, 12 women, average age 20.78 years, SD = 1.06) from the University of Electro-Communications. In the obtained response distribution, consensus was generally observed among the majority of participants for most of the images used in the cognitive test. However, some images exhibited variation in participant responses. Figure 5A illustrates the frequency of each option chosen for Image 2 with the least variability in responses. In contrast, Figure 5B depicts the responses of Image 11 with the most significant variability. In the case of Figure 5A, 91.3% of the young subjects selected “fuwa-fuwa,” serving as the basis for treating this option as the “correct” answer and assigning a high score to participants who chose this option. Conversely, in the scenario illustrated in Figure 5B, the choices “tsuru-tsuru” and “nuru-nuru” will be lower-as selected 29.0 and 46.4%, respectively.

In the distribution of responses from the healthy participant group, let $P\left(x_{j}|H_{i}\right)$ represent the proportion of choosing answer $x_{j}\left(1\leq j\leq 8\right)$ for image $H_{i}$ $\left(1\leq i\leq 12\right)$ . If a participant in the cognitive test selects answer $x_{n}$ $\left(1\leq n\leq 8\right)$ for the presented image, the assigned score is calculated using the following formula:

$$\mathrm{Score}\left(x_{n},H_{i}\right)=\frac{P\left(x_{n}|H_{i}\right)}{\max_{1\leq j\leq 8}P\left(x_{j}|H_{i}\right)}$$

For instance, consider an image where healthy individuals chose options 1 through 8 with proportions <0.5, 0, 0.1, 0, 0.2, 0.2, 0, 0>. If a participant selects $x_{3}$ as their response, the denominator in the formula would be 0.5, the numerator would be 0.1, resulting in a score of 0.2. The maximum score achievable for a single question is 1, while the minimum is 0. Using the above formula, the score for the whole cognitive ability test of the subject is determined by the sum of the scores for all questions. The maximum score a subject can obtain is 12 points, which is given when the combination of choices made by a subject for each image exactly matches the choices most frequently chosen among young healthy subjects. The minimum score is 0 points, which is given when the combination of choices made by a subject for each image completely aligns with choices that were never selected among young healthy subjects. In this study, the SSWTRT score would indicate the total score of 12 images.

### 2.4 Neuropsychological assessment

For the neuropsychological assessments, the Mini-Mental State Examination (MMSE), the Frontal Lobe Assessment Battery (FAB), and tests from the EU-iNPH grading scale were used; the RAVLT, the Grooved Pegboard, Stroop tests with color naming, and interference tests: which assess manual dexterity, verbal memory, psychomotor speed, and executive function. The aforementioned tests from the EU-iNPH Grading Scale were converted to scores in accordance with the conversation table from Hellström et al. (2012). Participants were then divided into two groups based on their MMSE scores. The entire neuropsychological assessment process took approximately 40 min to complete.

### 2.5 Statistics

Spearman’s correlation analysis was used to investigate the relationship between the SSWTRT test score, and the converted core for each neuropsychological test. The Welch’s *t*-test was used to compare between the two groups with regard to the SSWTRT, and the Mann–Whitney *U* test was used to compare between the two groups with respect to the neuropsychological tests.

A receiver operating characteristics (ROC) analysis was conducted to ascertain the potential of the SSWTRT scores to predict the cognitively normal group from the mild cognitive impairment group. The statistical analysis was completed through IBM SPSS version 29.0 for Macintosh.

### 2.6 Ethical approvals

This study was approved by the Research Ethic Committee of the Juntendo University, Tokyo, Japan (E22-0100). The preliminary experiment protocol was approved by the Research Ethics Committee of the University of Electro-Communications, Tokyo, Japan (#18026). The experiment was conducted between September 30, 2018, and March 31, 2019. The study adhered to the tenets of the Declaration of Helsinki and written informed consent was obtained from all participants including the preliminary experiment.

## 3 Results

The age and sex distribution of the 102 participants were age mean 77.9 (SD = 6.7, M: F, 54: 46). They visited Juntendo University Hospital and Juntendo Tokyo Koto Geriatric Medical Center from January to August 2023 and were either diagnosed as probable or definite iNPH by neurosurgeons and/or neurologists according to the Japanese iNPH Guidelines (Nakajima et al., 2021).

### 3.1 Result of study 1

Study 1 aimed to compare the SSWTRT with established neuropsychological tests. In Table 2 the averaged result of each image and the averaged total score of all 12 images are indicated.

**Table 2:** The result of SSWTRT scores.

	*n*	Mean	SD
Image 1	102	0.52	0.49
Image 2	102	0.75	0.43
Image 3	102	0.52	0.48
Image 4	102	0.36	0.36
Image 5	102	0.43	0.39
Image 6	102	0.57	0.4
Image 7	102	0.63	0.47
Image 8	102	0.58	0.37
Image 9	102	0.58	0.31
Image 10	102	0.6	0.4
Image 11	102	0.58	0.31
Image 12	102	0.51	0.38
Total	102	6.62	1.96

The scores for each image item score ranged from 0 to 1, with a minimum of 0.36 and a maximum of 0.75. The image with the highest score was Image 2 which indicates that significant proportion of patients had marked an SSW that would be chosen by the majority of healthy participants. The sum of each image would score up to 12, with a range of 1.7–11.4 and the mean score was 6.62 (SD = 1.96).

The neuropsychological test results are shown in Table 3, where the neuropsychological tests from the EU-iNPH grading scale are indicated in converted scores. The MMSE score ranged from a minimum of 15 to a maximum of 30 points. A number of patients were unable to complete the full set of tasks. This was either they had scored 0 for the conversion score, or they refused to do the tasks. Additionally, Table 4 presents the correlation between SSWTRT and the neuropsychological tests. A mild correlation was observed between the SSWTRT scores, and each of the neuropsychological test. The sum of the four neuropsychological scores of the EU-iNPH grading scale had the strongest correlation (*ρ* = 0.491, *p* < 0.001), followed by the converted score of RAVLT (*ρ* = 0.485, *p* < 0.001).

**Table 3:** The result of neuropsychological tests.

	*n*	Median	IQR 25–75
MMSE	102	26	23–28
FAB	95	14	13–16
EU-iNPH GS total score (pt)	81	240	180–295
Pegboard (pt)	84	60	30–80
RAVLT (pt)	89	50	30–80
Stroop color naming (pt)	90	50	30–70
Stroop interferes (pt)	86	75	50–90

**Table 4:** Correlation between the SSWTRT total score and the neuropsychological tests.

	MMSE	FAB	EU-iNPH GS total score	Pegboard (pt)	RAVLT (pt)	Stroop color naming (pt)	Stroop interference (pt)
SSWTRT Total score	0.443**	0.341**	0.491**	0.396**	0.485**	0.355**	0.471**

** *p* < 0.001.

### 3.2 Result of study 2

Individuals with an MMSE score of 28 points or above were categorized as having Normal Cognition (NC) group (*n* = 37). Those with MMSE scores between 22 and 27 points were considered to exhibit early cognitive decline and were designated as the Mild Cognitively Impairment (MCI) group (*n* = 50). Kaufer et al. (2008) used cut-off point of 27 or lower for the criteria for MCI. The name MCI is indeed used as a diagnostic symptom having different criteria. However, for the sake of familiarity, in this article, we will use the term MCI to indicate early cognitive decline according to the MMSE result.

**Figure 6:**
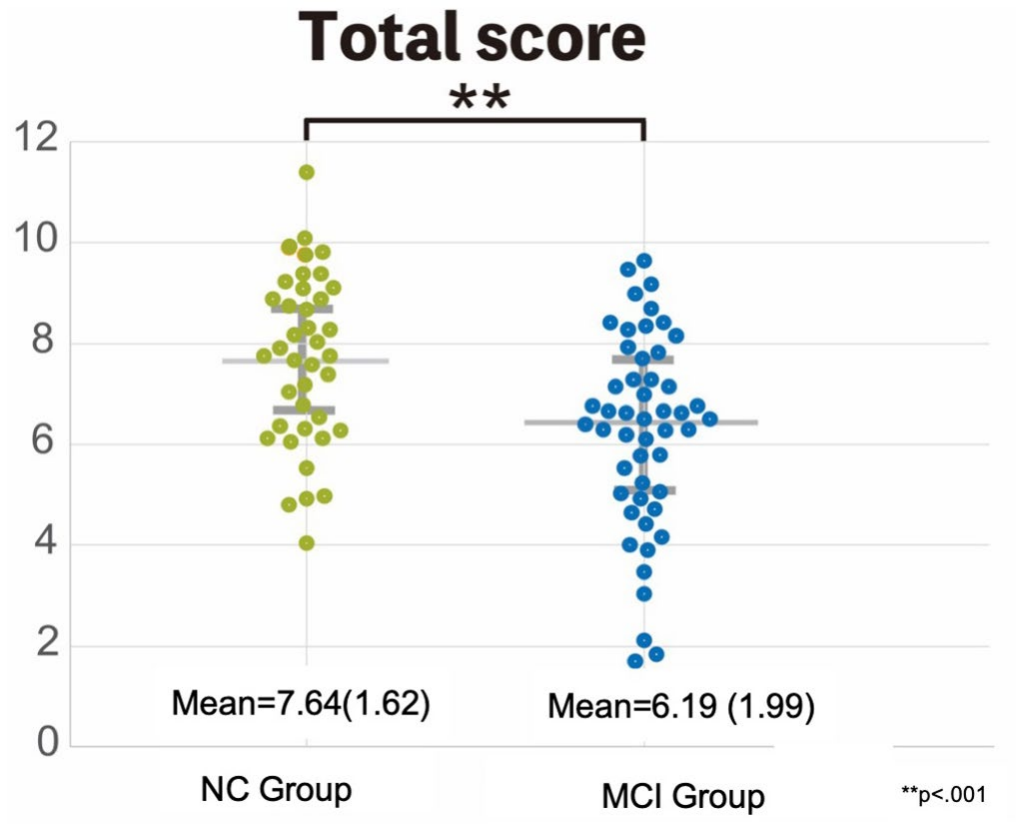
Comparison of the SSWTRT score between the two groups: the normal cognition and the MCI group. This graph shows the difference between the two grouped sound symbolic words texture recognition test (SSWTRT) total scores. The participants who had MMSE scores of 28 and higher were grouped as the normal cognition (NC) group. On the other hand, those who had MMSE scores between 22 and 27 were categorized as mild cognitive impairment (MCI) group. The total SSWTRT score was significantly greater in the NC group compared to the MCI group with a significance of *p* < 0.001.

The mean SSWTRT score for the NC group was 7.64 (SD = 1.62). On the other hand, the mean for the MCI group was 6.19, with an SD of 1.99. The mean and SD of each image and the group differences are indicated in Table 5. A comparison of the SSWTRT scores for each image between the two groups revealed significant differences for images 1, 2, 5, and 11, with *p*-values of *p* = 0.016, *p* = 0.008, *p* = 0.033, and *p* = 0.044, respectively. When focusing on the SSWTRT score, a significant difference was observed between the two groups. The distribution of the SSWTRT score is shown in Figure 6 with a significant difference of *p* < 0.001 between the NC group and the MCI group. A comparison of the neuropsychological tests between the two groups revealed significant differences in each test (see Table 6 for details).

**Table 5:** The comparison of the SSWTRT total score between two groups separated according to MMSE score.

	Normal cognitive group MMSE ≧ 28			MCI group 27 ≧ MMSE ≧ 22			
	*n*	Mean	SD	*n*	Mean	SD	*p*-value
Image 1	37	0.69	0.46	50	0.43	0.487	–
Image 2	37	0.89	0.312	50	0.66	0.472	–
Image 3	37	0.65	0.456	50	0.47	0.476	–
Image 4	37	0.39	0.359	50	0.39	0.377	–
Image 5	37	0.53	0.393	50	0.35	0.359	–
Image 6	37	0.63	0.374	50	0.57	0.394	–
Image 7	37	0.77	0.417	50	0.59	0.486	–
Image 8	37	0.69	0.336	50	0.55	0.368	–
Image 9	37	0.56	0.291	50	0.55	0.297	–
Image 10	37	0.67	0.396	50	0.53	0.413	–
Image 11	37	0.67	0.234	50	0.54	0.35	–
Image 12	37	0.51	0.369	50	0.56	0.38	–
Total	37	7.64	1.619	50	6.19	1.991	<0.001**

* *p* < 0.05, ** *p* < 0.001.

**Table 6:** Comparing the two MMSE scores with the neuropsychological tests.

	Normal cognitive group MMSE ≧ 28			MCI group 27 ≦MMSE≧22			
	*n*	Median	IQR 25–75	*n*	Median	IQR 25–75	*p*-value
FAB	37	15	15–16	46	13	13–15	<0.001**
Pegboard (point)	35	80	50–90	40	40	30–70	<0.001**
RAVLT (point)	35	80	50–90	44	40	30–60	<0.001**
Stroop color naming (point)	35	60	50–90	44	50	22.5–60	<0.001**
Stroop interference (point)	35	90	70–100	44	60	40–80	<0.001**
iNPH GS total	35	280	260–360	40	215	142.5–247.5	<0.001**

* *p* < 0.05, ** *p* < 0.001.

**Figure 7:**
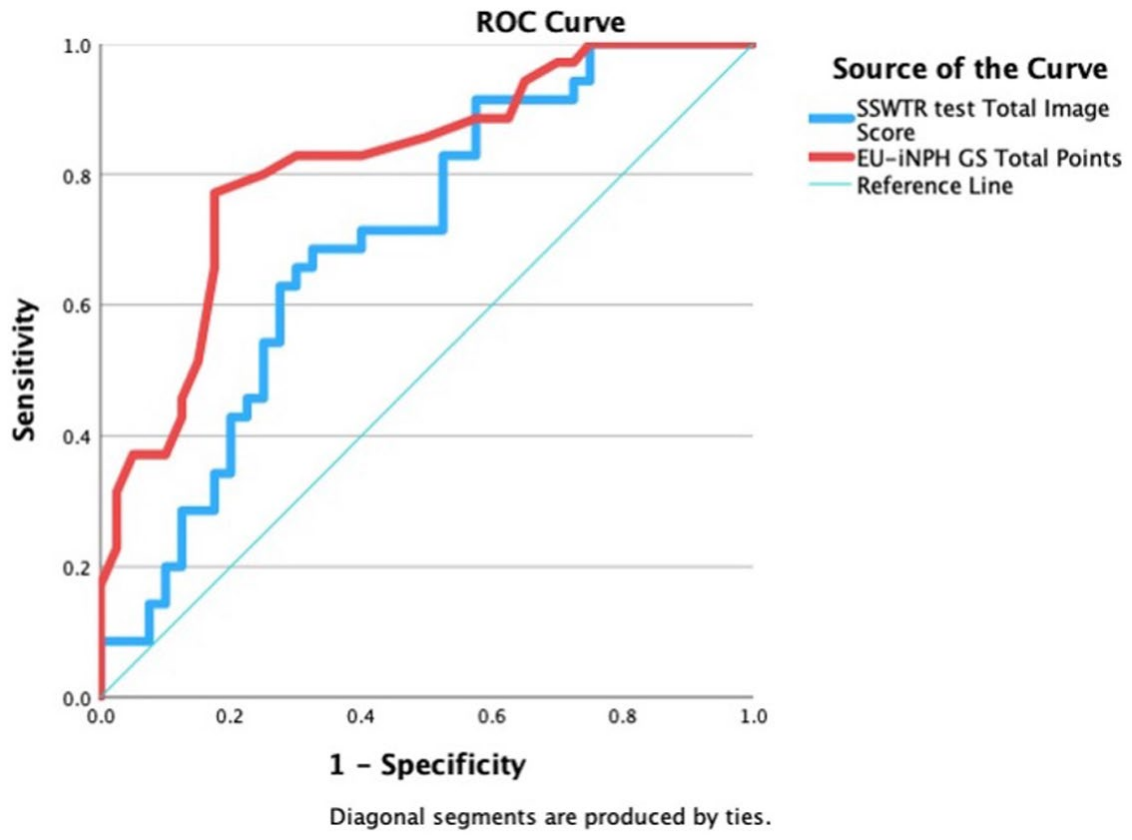
The result of ROC analysis of SSWTRT total score. Receiver operating characteristics curves (ROC) for predicting mild cognitive impairment with the total score of the sound symbolic words texture recognition (SSWTRT) test, The ROC analysis was used to evaluate the ability of the SSWTRTT score to differentiate the two groups, those with normal cognition (NC) and those with a Mild Cognitive Impairment (MCI). The two groups were separated according to the MMSE score. Patients with MMSE of above 28 were considered as the NC group, and those with MMSE between 22 and 27 were considered the MCI group. The SSWTRT score is the sum of the score of 12 images. The participants were asked what they would feel if they were touching the material shown in the photo and answered by choosing the best SSW from 8 choices. The SSWTRT score predicted the two MMSE-separated groups with an AUC of 0.703 which is shown as a blue line in the graph with sensitivity of 68% and specificity of 68%. The ROC analysis was also done with the iNPH-GS (grading scale) converted score to calculate the predictive power of the grading scale. EU-iNPH GS is constructed from four neuropsychological tests and those results are converted into scores from 0 to 100. iNPH GS total score is the sum of all four neuropsychological tests. It too was able to predict the difference between the two groups separated by MMSE with AUC of 0.815, sensitivity of 77% and specificity of 83%.

To assess the predictive value of the SSWTRT score, the ROC curve analysis was conducted. This analysis demonstrated that the SSWTRT score can effectively differentiate between the two groups, the NC group and the MCI group, having a cut-off value of 7.01 with a sensitivity of 68% and Specificity of 68%, and area under the curve (AUC) of 0.708. The results of the ROC curve are shown in Figure 7. A further ROC analysis was conducted to enable a comparison with the SSWTRT score. The sum of the results of the EU-iNPH grading converted scores was used to analyze whether it can differentiate the patients into two groups, the NC group and the MCI group. The total score of the four neuropsychological tests from the EU-iNPH grading score was found to be able to differentiate the two groups through a cut-off value of 255, with a sensitivity of 77% and specificity of 83%, and an AUC of 0.815.

## 4 Discussion

In this study, we have created a new cognitive test, the SSWTRT, which is capable of detecting cognitive decline in patients with iNPH by evaluating their ability to recognize texture and expressing it in SSW. The validity of the SSWTRT was evaluated by comparing its results with those obtained from neuropsychological tests from the EU-iNPH grading scale which includes memory and learning, hand dexterity, psychomotor speed, and executive function in which iNPH patients tend to decline.

As hypothesized, the SSWTRT demonstrated a mild correlation with the existing neuropsychological tests, including the Pegboard, RAVLT, and the two tasks of the Stroop test. The results indicated that patients with iNPH and mild cognitive decline exhibit difficulty in recognizing image texture than those with normal cognitive function. In addition to having a correlation with MMSE and FAB, the texture recognition test showed a mild correlation with the total score of neuropsychological tests and RAVLT and from the iNPH grading scale.

The test battery in the EU-iNPH grading scale is designed to assess memory and learning, hand dexterity, psychomotor speed, and executive function. Saito et al. (2011) have reported the relative proportions of impairment in individual cognitive domains of iNPH. In the aforementioned study, the relative proportions of impairment in individual cognitive domains of iNPH were as follows: 52.1% for executive function, 41.8% for episodic memory, and 6.1% for visuoperceptual/visuospatial function in iNPH patients (Saito et al., 2011). The SSWTRT was found to have a potential of becoming an effective tool for detecting cognitive changes associated with iNPH. It can be assumed that the process of texture recognition and SSW expression necessitates the integrated function of the brain, which encompasses visual perception, memory, and verbal learning.

The SSWTRT does not actually require the patients to touch the object, but rather it requires them to recall from their past experience what would be the texture of the surface in the picture they would feel if they had touched it. As Oishi (2024) states that texture recognition is learned from individual’s experiences. For texture recognition, the visual information is processed through the ventral visual pathway, and retrieving the information from the memory to determine a kind of surface it is. In the ventral visual stream, simple visual characteristics are informationally transformed into psychological impressions, and the activity in the more ventral higher-order visual areas is closely related to material recognition (Kaufer et al., 2008). Such information would be transmitted to the language areas to match the closest word in SSW that they have learned throughout their lives. Buckingham et al. (2009) and Gallivan et al. (2014), who have reported the estimation of object perception through visual appearance, emphasized that the link between material perception and object weight estimation is empirically relevant (Saito et al., 2011; Guerrero-Berroa et al., 2009). The process of texture recognition and its representation through SSW is considered to be a multimodal process in which different functions of the brain are interrelated (Oishi et al., 2018; Kaufer et al., 2008). Therefore, having correlation with cognitive tests, such as RAVLT that measures verbal learning and recall would indicate that those who retain memory function are likely to answer the SSWTRT correctly since they are successful in retrieving information from their past experience.

It is not uncommon for neuropsychological tests to be perceived as a burden by patients. One of the reasons for this is that it is a time-consuming process. In creating the MMSE, administration time was considered as one of the important factors when evaluating the cognitive function of the elderly. He therefore constructed the test to be administered within 5–10 min (Folstein et al., 1975). As cognitive function declines with the increase of test duration, additional time is required for the administration of the test (Guerrero-Berroa et al., 2009). The neuropsychological tests from the EU-iNPH grading scale have four different tasks included, requiring approximately 40 min to administer. In the Juntendo University Hospital, all the neuropsychological tests were conducted within an hour of the reservation slot, including a time for rapport building through small talk and giving feedback on the test results. The SSWTRT, similar to the MMSE, took approximately 10 min to complete including the description of the administrative procedure to the patients. It would be beneficial to both patients and administrators if the test times are shortened. For the patients, this would result in a reduction of stress associated with the administration of the tests. For the test administrators, a reduction in the time spent on one patient would allow the tester to take another patient within a specific timeframe with all consequent associated organizational and economic benefits.

The SSWTRT could be beneficial in a way that the answers to the questions are not explicitly obvious compared to the existing neuropsychological tests, which would make patients less likely to feel that they are being judged by the test. Most of the neuropsychological tests have clear answers that the patients are likely to know without special knowledge. Often, the patients would feel uncomfortable or even embarrassed if they knew they could not answer such an “easy” question. These are questions that the patients would probably have been able to answer in their healthy state. The neuropsychologists have worked seriously to prevent the patients from feeling stress by the neuropsychological test by establishing a rapport when administrating the test. The process of establishing rapport through small talk or explaining the purpose of the test is very important to make the patient feel comfortable about taking the test, but it takes time and professional training to be successful in it. Avoiding the patients to be in an embarrassing situation by not being able to answer simple questions would lead to protecting the dignity of patients from exposing their cognitive decline in front of other people, such as the examiner. Patients with dementia often experience self-stigma and have ambivalent feelings about being diagnosed with dementia (Bhatt et al., 2021).

Another benefit of not having a clear answer is that it avoids the learning effect of repeated administrations. To evaluate the effect of an intervention it is often necessary to administer the cognitive tests in a relatively short period of time. Even if the patients do not remember exactly what they will be asked in the neuropsychological tests, they have clear expectations. Although the effect of such predictability needs further investigation, in the case of the SSWTRT, even if the patients can predict what will be asked to do, the result is less likely to be affected by the previous administration because they would not know which choice of the SSW was correct.

Lastly, the SSWTRT would not require the assistance of a professional to administer. The administration procedure of commonly used cognitive tests such as the MMSE may be simple, but it is difficult to be administered by the patients alone. The questions and procedures of the MMSE are not very difficult, but professional training is required for its administration. MMSE has a defined necessary step in giving instructions and directions to the patients and how it is handled by the patients is also observed by the professionals. On the other hand, the SSWTRT only requires only a single type of task for the patients. Its administration procedure is given to the patients in written form and the patients only need to follow the instructions, they do not even need to write anything down. In addition, the test pages are designed so that they intuitively can tell what to do at a glance since the SSW is commonly used in everyday life and only the SSW used for texture is included in the SSWTRT. Allowing non-trained professionals to administer the SSWTRT may be one of the solutions to the shortage of neuropsychologists, especially in Japan. Also, the SSWTRT could be administered in homes or in institutions where elderly people spend their day. In addition, the scoring of the SSWTRT is unique in a way that it is not decided in an all-or-nothing format. This means that the patients do not necessarily need to score a full point to pass the cut-off point of 7.01, allowing patients to make minor mistakes or accept slight differences that may be caused by educational or cultural differences.

The SSWTRT, which only takes approximately 10 min to administer without a professional’s help, could be useful to those who are reluctant to go to hospitals but have cognitive dysfunction characteristics that are noticed by people around them. Since the SSWTRT has a mild correlation with the EU-iNPH GS, those with low SSWTRT score would show impairment in their cognitive abilities. The SSWTRT can be used to bring those people in the early stages of dementia to a hospital and provide the necessary treatment at an early stage of their symptoms for it is well known that the prognosis would differ depending on when the treatment started.

## 5 Limitations

This study has several limitations. First, we have only included patients with iNPH in a representative group of cognitive decline. Many types of diseases show symptoms of cognitive decline, but this study only included iNPH patients. It is also important to consider that within iNPH, it is common to have comorbidities such as iNPH with Alzheimer’s disease or Parkinson’s syndrome. Each comorbidity would show different characteristics of cognitive decline, where iNPH with Alzheimer’s disease comorbidity would have dysfunction in language learning and memory, and iNPH with Parkinson’s syndrome would tends to have dysfunction in psychomotor speed and executive functions. For further study, it is necessary to seek the relationship of the SSWTRT with other types of dementia. Second, the mechanism of texture recognition and the use of SSW was not clarified in this study. In further research, the use of tools such as functional imaging techniques would be necessary to visualize the process of information processing in the brain while people recognize the texture, and how it is transformed into SSW. Third, this study was limited in scope, focusing exclusively on the use of SSW in the context of Japanese. Given that the SSW employed differs between languages, it is necessary to examine the efficacy of the SSWTRT in other languages, such as English. The study by Wong et al. (2022, p. 22) examined the cross-cultural and cross-linguistic similarity between Japanese SSWs and the perception of the hardness and softness of objects. The Singaporean and US participants were included in the study, and their judgments of “hard” and “soft” words were found to be similar to those of Japanese participants. Specific letters were found to be related to the perception of the hardness or roughness of an object regardless of cultural and linguistic backgrounds. Further research would be beneficial in order to create SSWTRT that could be used universally.

## 6 Conclusion

In conclusion, we have created the SSWTRT that reflects the results of neuropsychological tests of cognitive deterioration and differentiates early cognitive decline in patients with iNPH but is easier to administer and consists of only 12 similar image tasks. Compared to the current neuropsychological tests, the SSWTRT is expected to be performed in a shorter time, which would result in less burden for the patients. The SSWTRT could be used as a screening to detect mild cognitive decline in iNPH.

## Data Availability

The raw data supporting the conclusions of this article will be made available by the authors, without undue reservation.
